# Audit of physical health monitoring during initiation and ongoing treatment with antipsychotic medication in a tier 3 outpatient CAMHS service, Belfast

**DOI:** 10.1192/bjo.2021.851

**Published:** 2021-06-18

**Authors:** Pam Hamlyn, Aaron McMenamin, Hilary Boyd, Lara Patton

**Affiliations:** Belfast HSCT

## Abstract

**Aims:**

To evidence that physical health monitoring during antipsychotic initiation and continued treatment within the Child and Family Clinic is current, as per the agreed Antipsychotic Medication Monitoring Schedule for Belfast Trust CAMHS (2015), supporting Quality Network for Community CAMHS(QNCC) accreditation.

**Background:**

The Antipsychotic Medication Monitoring Schedule CAMHS(2015) was agreed by a working group of consultant psychiatrists and pharmacists, based on evidence from The Canadian Alliance for Monitoring Effectiveness and Safety of Antipsychotics in Children (CAMSEA), NICE Guidelines CG 185(2014), CG155(2013) and Maudsley Guidelines, and was to be located on the electronic system (PARIS).

**Method:**

In January 2019, a list of all children/young people on antipsychotic medication was collated (n = 12). Presence of the monitoring schedule in the clinical notes or PARIS was recorded. The Electronic Care Record was reviewed for blood results and PARIS letters for documentation of physical health parameters (heart rate, blood pressure, height, weight, BMI, extrapyramidal side effects, ECG) and to identify documentation of risk/benefit review where monitoring was declined. Re-audit January 2020 (n = 9). Criteria:

All patients commenced on antipsychotic medication will have baseline blood investigations and other physical health parameters documented as per the monitoring schedule. If monitoring was declined, the reason for this and indications for prescribing must be documented as a risk/benefit analysis.

All patients on antipsychotic medication will be current with their physical health Monitoring Schedule.

All patients will have their Monitoring Schedule completed in clinical notes or on PARIS.

**Result:**

First cycle results (n = 12):

Baseline bloods (or documented declined) = 92%, Baseline ECG (or documented declined) = 75%

Complete monitoring bloods = 33%, Physical health monitoring parameters complete = 42%

Monitoring schedule present in the notes and current = 42% (0% on PARIS).

Initial Recommendations: Standardised recording of monitoring using PARIS clinic letters and the schedule in front of clinical notes; Baseline ECG mandatory

Second cycle results (n = 9):

Baseline bloods (or declined) = 89%, Baseline ECG (or declined) = 67%

Complete monitoring bloods = 44%, Physical health monitoring parameters complete = 56%

Monitoring schedule present in notes and current = 38%, Present, not current = 50% (0% on PARIS).

**Conclusion:**

Lower numbers at re-audit limit interpretation.

Further recommendations: Antipsychotic initiation checklist; Central bloods diary for clinicians; Antipsychotic care-pathway booklet, co-produced with young people, incorporating the monitoring schedule.

